# Effects of *Moringa oleifera* on working memory: an experimental study with memory-impaired Wistar rats tested in radial arm maze

**DOI:** 10.1186/s13104-022-06219-5

**Published:** 2022-10-03

**Authors:** Sadia Afrin, Ahmed Hossain, Shelina Begum

**Affiliations:** 1 Department of Physiology, Marks Medical College and Hospital, Dhaka, Bangladesh; 2 College of Health Sciences, University of Sharjah, Sharjah, United Arab Emirates; 3 Department of Public Health, North South University, Dhaka, Bangladesh; 4 Department of Physiology, Bangabandhu Sheikh Mujib Medical University, Dhaka, Bangladesh

**Keywords:** Memory, *Moringa oleifera*, Working memor*y*, Radial arm maze test, NMDA receptor

## Abstract

**Objective:**

Memory impairment is a serious problem that has a significant negative impact on survival and quality of life. When used for a long time, drugs used to treat memory loss become less effective and have more side effects, making therapy more difficult. Different medicinal plants are now being highlighted because of their valuable applications and low risk of adverse effects. *Moringa oleifera* is one of these plants that has gained much attention due to its diverse biological functions. The study aimed to determine the effects of *Moringa oleifera* on working memory in memory-impaired Wistar rats.

**Results:**

For this experimental study, 30 male Wistar rats having 150–250 g bodyweight were divided equally into three groups: Group-I/normal memory group (treated with oral normal saline 5 ml/kg body weight), Group-II/memory-impaired group (induced by intraperitoneal ketamine 15 mg/kg body weight), and Group-III/experimental group (treated with oral *Moringa oleifera* 200 mg/kg bodyweight and intraperitoneal ketamine 15 mg/kg body weight). The experimental group showed significantly fewer working memory errors than the memory-impaired group. The experimental group also provides the lowest variability of WMEs among groups. Thus, the study concludes that *M. oleifera* can prevent ketamine-induced memory impairment in Wistar rats.

**Supplementary Information:**

The online version contains supplementary material available at 10.1186/s13104-022-06219-5.

## Introduction

Humans can adapt their behavior based on past experiences because of their ability to retain information [1]. Memory impairment is a characteristic of dementia that has been declared a global challenge. Dementia is a severe loss of cognitive ability, including memory impairment. Owing to the rapid growth in prevalence, high expenditure cost, and unsatisfactory outcomes of therapeutic strategies, dementia has been recognized as a significant medical and social challenge, especially in developing countries. 46.8 million people live with dementia worldwide, reaching around 74.7 million in 2030 and 131.5 million in 2050, almost doubling every 20 years [2].

Memories can be classified into short-term, intermediate, and long-term memory based on the time of storage [3]. This short-term memory is referred to as working memory (WM), and long-term memory is referred to as reference memory [3, 4]. WM refers to a system of our brain that provides temporary storage and manipulation of information [5]. The WM allows temporal storage of a limited amount of spatial information, the geographic information related to a specific location. Data stored in WM can be actively maintained for a short time and then rapidly forgotten or stored elsewhere in the brain as long-term memory [6–9].

Neural activity is necessary for WM. Different receptors and channels have been reported to be involved in memory formation, such as the muscarinic receptor, nicotinic receptor, *N*-methyl-d-aspartate (NMDA) receptor, 5HT1Areceptor, D1receptor, Ryanodine receptor, etc. [10, 11]. However, in the last two decades, the role of the NMDA receptor has been reported in the development and maintenance of WM [12–14]. In WM, the NMDA receptor is associated with persistent neural activity [13, 15]. Blocking NMDA receptors (NMDAR) might cause WM impairment [13]. Ketamine is a noncompetitive NMDAR antagonist that interacts with NMDA receptors to exert its effects for action [16–18]. It might inhibit persistent neural activity, interfering with memory formation [13, 18]. Different investigators reported a significantly impaired memory in animal models after administration of intraperitoneal ketamine [18–20].

Memory impairment does not have a definitive treatment. Most of the drugs administered so far aim to treat the pathophysiology of disorders that cause memory loss. Most medications, such as antipsychotics and cholinesterase inhibitors, have limited efficiency due to long-term use and substantial side effects from non-selective action on numerous organs, making treatment more challenging.

Herbal items could be utilized to mitigate the side effects of certain medications used to treat memory impairment. Among many herbal products, *Moringa oleifera* [Family: Moringaceae] could be an important medicinal herb [21–24]. It is commonly known as “Sajna” in Bengali, used as a vegetable, spice, cooking and cosmetic oil source, and medicinal plant [25]. Almost every part of this plant, including root, bark, gum, leaf, flowers, and seeds, is functional, and hence it is named a “Multipurpose tree” [26]. Different parts of the plant are a good source of proteins, vitamins A, B, and C, minerals, beta carotene, amino acids, flavonoids, saponins, phytates, and various phenolic constituents, which act as antioxidants [26]. The neuroprotective and cognitive boosting may be benefitted in part by the flavonoids of *M. oleifera* leaf extract. We investigate the effects of *Moringa oleifera* on WM in memory-impaired Wistar rats.

## Materials and methods

### Subjects

We took male Wistar rats for the study because it was suggested that sex hormones could influence learning memory performance in rats [27]. Thirty rats weighing 150 to 250 g were obtained from the Bangabandhu Sheikh Mujib Medical University's (BSMMU) animal house. All of the rats were housed in specially constructed plastic cages in the Department of Physiology's rat facility. The rats were returned to BSMMU's rat laboratory when the experiment was completed.

### Grouping

Thirty rats were randomly divided into three equal groups: Group I/normal memory group (treated with oral normal saline 5 ml/kg body weight), Group II/memory impaired group (induced by intraperitoneal ketamine 15 mg/ kg body weight), and Group III/experimental group (treated with oral *M. oleifera* 200 mg/kg body weight and intraperitoneal ketamine 15 mg/kg body weight). An 8-arm typical radial maze made of plexiglass was utilized in the experiment, and a description can be found in Additional file 1.

### Plant materials and preparation

The *Moringa oleifera* leaves was obtained from the field of Bangladesh Council of Scientific and Industrial Research (BCSIR), Dhaka. The process of ethanolic extraction of the leaves was adapted from Mahaman et al. [27]. The plant leaves were picked and washed with fresh water before being dried in the sun for a week. A mixer grinder was used to smash the leaves, which were then stored in an airtight container until needed. The powdered leaf sample was then extracted with 95% ethanol in an orbital shaker. The powder was steeped in 1000 ml of 95% ethanol for 200 g. Continuous stirring was used to obtain the extract, which was kept at room temperature for 2 days. The extract was then filtered with a cotton plug to get rid of plant debris, and afterward through Whatman filter paper several times. Finally, it was concentrated using a vacuum rotary evaporator at 60 °C. A detailed extraction procedure is given in Additional file 1.

### Procedure

Ten rats from each group were acclimatized for 7 days at the animal lab for the RAM test. The training was done in 3 phases: habituation/shaping (6 days), acquisition (5 days), and retention (7 days). During all the phases, every day every rat was brought into the memory lab for trials (trial 1 and trial 2) separated by 3 h. In this test, a fasting rat had to search for food. For this, before the beginning of trial 1, each rat was deprived of only food (not water) for approximately 10 h. Trial 1 was started 30 min after administering the prefixed treatment based on group assignment. After each test, the maze was thoroughly cleaned with 70% alcohol to minimize residual odor. Three days before starting habituation, the rat was introduced to the bait/ food pellet in the rat cage once every day.

#### Habituation/shaping phase

On the 1st day of habituation (Day 16), two rats at a time were put in the maze with baits for a 10 min trial. The next day (Day 17), each rat was given unique access with the same baiting as the previous day. For the RAM test, these 2 days were used as reference memory arms. Only the eight-food cup in the maze's eight arms was baited on the third and fourth days (Days 18 and 19). However, on the last 2 days (Days 20 and 21), any four arms were baited at random (by lottery). In each trial, the baiting arm numbers differed between rats.

#### Acquisition phase

During the acquisition phase, any four of the eight arms were baited by jilapi in a food cup. Each trial was started by placing a rat at the center of the platform with all gates closed. Then all gates were opened at a time. When the rat entered any one arm, the seven other gates were closed. After exploration, the rat came out, and its gate was locked. All gates were opened again after 5 s, and the whole process was repeated. The trial was continued for 10 min or all jilapis of the four baited arms were eaten by the rat, whichever occurred first. The trial-two was repeated in the same manner for each rat after 3 h.

#### Retention phase

During the retention phase, the rat was kept in its cage without any training but with a daily dose of supplementation. On day 33 (the seventh day after the acquisition phase), a retention test (comprising two trials) was performed following the acquisition phase technique.

### Outcome measures: WMEs

The four arms of the RAM test were chosen by random from eight arms that were baited with meals for each rat. The rat's return to those four arms was seen as working memory errors (WMEs) [28]. Thus, WMEs are indicated by re-entries into arms that have already been used for bait during a testing session and re-entries into reference memory arms.

### Statistical analysis

Data were expressed as mean of variables ± standard error for WMEs. We investigated the heterogeneity in WMEs by different groups and provided the significant differences among the groups when days and trials are considered. The ANOVA followed by Bonferroni post hoc test were used using SPSS (Version 16). To find the effect of *M. oleifera* on WMEs, p ≤ 0.05 was considered significant.

## Results

Table 1 provides the mean of WMEs and standard errors (SEMs) of mean WMEs by groups, experimental days, and trials. The mean ± SEM of WMEs were 2.80 ± 0.36, 3.40 ± 0.70, 2.20 ± 0.29 frequency/trial in trial 1 and 1.70 ± 0.39, 3.80 ± 0.36, and 1.30 ± 0.33 frequency/trial in trial 2 in group I, II, and III, respectively, on day 22. It appears that group II provides a high variability in values of WMEs than groups I and III. From days 22 to 26 and on day 33, the mean WME was different across all groups for both trials, except for trial 1 on day 22, trial 1 on day 23, and trial 2 on day 24. Furthermore, the mean WME in group II was higher compared to group I from days 22 to 26 and day 33 for both trials, except day 22 for trial 1, day 23 for trial 1, day 24 for both trials, and day 33 for trial 1. Also, from days 22 to 26 and day 33, the mean WMEs of this variable were significantly lower in group III compared to group II for both trials (trial 1 and trial 2), except for trial 1 on day 22; trial 1 on day 23; and trial 2 on day 24. The results indicate that the heterogeneity of WMEs in group II is greater than in groups I and III, whereas the experimental group has the lowest variability of WMEs.

**Table 1:** Working memory errors (frequency/trial) on different days of RAM test in various groups of rats

Phases	Experimental days	Trials	Groups		
			I	II	III
Acquisition phase	Day 22	T1	2.8 ± 0.36 (1 to 4)	3.40 ± 0.70 (0 to 7)	2.20 ± 0.29 (1 to 3)
		T2	1.70 ± 0.39 (0 to 4)	3.80 ± 0.36 (2 to 6)	1.30 ± 0.33 (0 to 3)
	Day 23	T1	2.00 ± 0.29 (1 to 4)	2.40 ± 0.49 (0 to 5)	1.90 ± 0.17 (1 to 3)
		T2	1.00 ± 0.25 (0 to 2)	2.20 ± 0.44 (0 to 4)	0.90 ± 0.23 (0 to 2)
	Day 24	T1	2.00 ± 0.29 (1 to 3)	2.60 ± 0.26 (1 to 4)	1.50 ± 0.16 (1 to 2)
		T2	1.20 ± 0.24 (0 to 2)	1.90 ± 0.31 (0 to 4)	1.10 ± 0.17 (0 to 2)
	Day 25	T1	1.40 ± 0.22 (1 to 3)	2.80 ± 0.51 (0 to 5)	1.20 ± 0.20 (0 to 2)
		T2	0.50 ± 0.22 (0 to 2)	1.90 ± 0.27 (1 to 3)	0.40 ± 0.16 (0 to 1)
	Day 26	T1	0.80 ± 0.13 (0 to 1)	2.30 ± 0.44 (0 to 4)	0.70 ± 0.16 (0 to 1)
		T2	0.40 ± 0.22 (0 to 2)	2.20 ± 0.35 (0 to 4)	0.30 ± 0.15 (0 to 1)
Retention day	Day 33	T1	1.40 ± 0.22 (0 to 2)	2.60 ± 0.52 (1 to 6)	1.20 ± 0.20 (0 to 2)
		T2	0.50 ± 0.22 (0 to 2)	1.80 ± 0.33 (0 to 3)	0.40 ± 0.16 (0 to 1)

Each column symbolizes mean ± SEM for 10 rats. Values in parenthesis indicate ranges. Ia = rats with oral normal saline (5 ml/kg) for consecutive 26 days (day 8 to day 33); IIa = rats with intraperitoneal (IP) ketamine (15 mg/kg) on each day of acquisition phase for consecutive 5 days (day 22 to day 26); IIIa = rats with oral *Moringa oleifera* (200 mg/kg) for consecutive 26 days (day 8 to day 33) and IP. ketamine (15 mg/kg) for consecutive 5 days of the acquisition phase (day 22 to day 26). RAM = radial arm maze

**Fig. 1:**
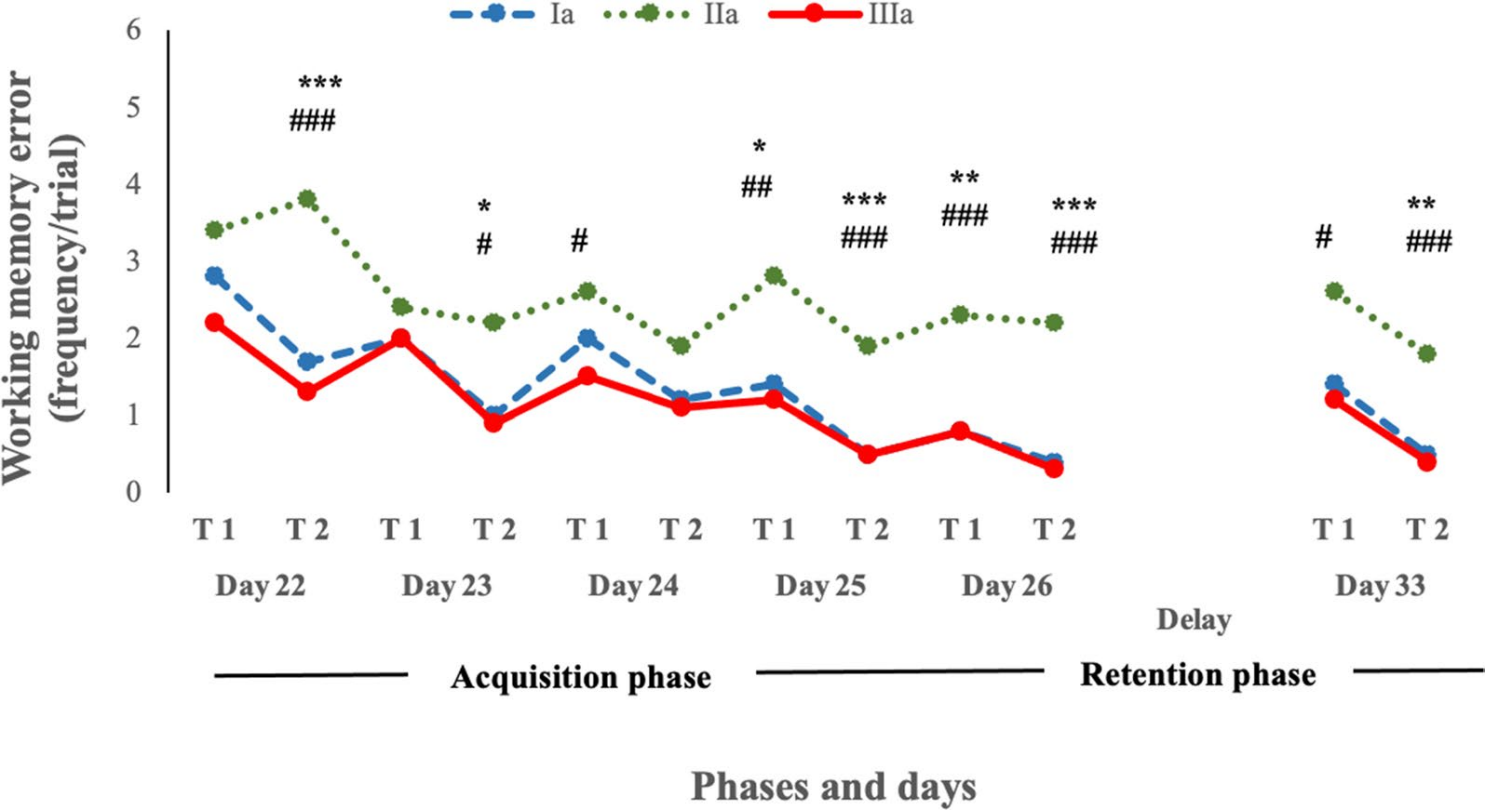
Working memory error in different trials of different days of RAM test in different groups of rats (Note: *Ia vs IIa, ^$^Ia vs IIIa, ^#^IIa vs IIIa. ^*/#/$^p ≤ 0.05; ^**/##/$$^p ≤ 0.01; ^***/###/$$$^p ≤ 0.001)

Figure 1 presents the WMEs in different trials. Each line symbolizes the mean WMEs for ten rats in each experimental group. The findings suggested a significant difference between II and I/III at various times. Overall, the III provides a minimum WMEs, which suggests the experimental group, rats with oral *M. oleifera* (200 mg/kg) for consecutive 26 days (day 8 to day 33) and ketamine (15 mg/kg) for five consecutive days of the acquisition phase (day 22 to day 26) provides a better result for improving memory. It also appears that group III had significantly lower WMEs than group II at each trial and day.

## Discussion

The study evaluated the effects of *M. oleifera* on ketamine-induced memory-impaired male Wistar rats and NMDA receptors. For this, ketamine-induced memory-impaired male Wistar rats were studied to observe the effects of the medicinal herb on WM.

We used ketamine to impair memory in our memory-impaired group of rats*,* as evidenced by significantly increased WMEs in the RAM test compared to the normal memory group. Many studies reported significantly impaired memory in animal models after administration of intraperitoneal ketamine [22, 24–26]. Quercetin, one type of flavonoid of M. olivera, was found to significantly increase the expression of NR2A and NR2B subunits of NMDARs. A study found that omega-three polyunsaturated fatty acid, a component of MO leaves, increased the NR2B subunit in the prefrontal cortex and hippocampus [26]. Some researchers also showed that the dietary polyunsaturated fatty acid of *M. oleifera* increased NR2A and NR2B subunits expression in the hippocampus [26, 27]. This may explain why increasing subunits of NMDARs might cause the prevention of working memory impairment in the experimental rats with *M. oleifera*.

We found a sub-anesthetic dose of ketamine (15 mg/kg) caused memory impairment in ketamine-induced rats. This impairment may be due to the blockade of NMDA receptors in the postsynaptic membrane of the pyramidal neuron of the prefrontal cortex [29]. As a result, persistent neural activity among pyramidal neurons of PFC, which is essential for maintaining WM, might get hampered.

In the experiment, *M. oleifera* was found to reduce significantly WMEs. A study found that a component of *M. oleifera* leaves, omega-3 polyunsaturated fatty acid, enhanced the NR2B subunit in the prefrontal cortex and hippocampus [30]. In the hippocampus, dietary polyunsaturated fatty acids from *M. oleifera* boosted the expression of NR2A and NR2B subunits [31]. As a result, increasing the number of NMDAR subunits in the experimental rats may prevent WMEs. Ketamine, on the other hand, is a noncompetitive NMDAR antagonist, and the majority of its actions are transmitted by its interaction with NMDA receptors [23, 32–34].

## Conclusion

According to the findings, *Moringa oleifera* can prevent ketamine-induced memory impairment in male Wistar rats, and NMDA receptors may be implicated in this preventive effect of *Moringa oleifera*. Another study with a specific NMDA receptor blocker employing a computerized instrument should be investigated for additional investigation to confirm our findings.

## Limitations

There are some limitations to the study. The eight-arm radial maze's WM component may be more difficult for some rats to learn than other maze problems. Despite these small drawbacks, using an eight-arm RAM to examine working and reference memory throughout the animal model is a reliable method. Limited samples in each group may fail to include the heterogeneity of the data. Another limitation is we did not consider the fourth group (treated with oral *M. oleifera* 200 mg/kg body weight) for investigation. As a result, it is critical that this knowledge be shared with the scientific community to encourage more study that builds on our findings.

## Supplementary Information

**Additional file 1: S1.** Justification of sample size. **S2.** Radial arm maze. **S3.** Components of extract and standardization. **S4.** Plant identification, location of where they were gathered. **S5.** Process of ethanolic extraction of *Moringa oleifera* leaves.

## Data Availability

The dataset of the current study is available from the corresponding author on a reasonable request.

## Abbreviations

RAM: Radial arm maze
*M*. *oleifera*: *Moringa oleifera*
*WM*: Working memory
*WME*: Working memory error
NMDA: *N*-methyl-d-aspartate
