# Supplementary material for: Effects of Moringa oleifera on working memory: an experimental study with memory-impaired Wistar rats tested in radial arm maze
Source: BMC Res Notes. 2022 Oct 3;15:314. doi: 10.1186/s13104-022-06219-5 (PMC9528094; doi:10.1186/s13104-022-06219-5)
Supplement: Supplementary file 1 — Additional file 1: S1. Justification of sample size. S2. Radial arm maze. S3. Components of extract and standardization. S4. Plant identification, location of where they were gathered. S5. Process of ethanolic extraction of Moringa oleifera leaves. [file 13104_2022_6219_MOESM1_ESM.pdf]

S1. Justification of sample size

As the design of this study was experimental, here specified difference between two means of WMEs to test the null hypothesis. So, following formula (Kirkwood and Sterne, 2003) involving effect size was used to calculate the intended sample size for a group (either study or control):

$$n = [(u+v)^2 \times (\sigma_1^2 + \sigma_2^2)] / (\mu_1 - \mu_2)^2$$

Level of significance, u = 1.96 (At 5% level)

Power of the test, v = 0.85 (At 80% of power)

Mean of WMEs for experimental group, μ1 = 67

Mean of WMEs for control group, μ2 = 75

SD of WMEs for experimental group, σ1 = 4.9

SD of WMEs for control group, σ2 = 7.34 (Sutalangka et al. 2013)

Therefore,

$$n = [(u+v)^2 \times (\sigma_1^2 + \sigma_2^2)] / (\mu_1 - \mu_2)^2$$

$$= [(1.96 + 0.85)^2 \times (4.9^2 + 7.34^2)] / (67 - 75)^2$$

$$= [7.896 \times (24.01 + 53.93)] / 8^2$$

$$= [7.896 \times 77.94] / 64$$

$$= 615.42 / 64$$

$$= 9.62$$

= 10 for any one group

Therefore, we utilized n = 10 3 = 30 rats because the total number of groups in this study was three.

Sutalangka C, Wattanathorn J, Muchimapura S, Thukham-mee W. Moringa oleifera mitigates memory impairment and neurodegeneration in animal model of age-related dementia. Oxid Med Cell Longev. 2013;2013:695936. doi: 10.1155/2013/695936. Epub 2013 Dec 23. PMID: 24454988; PMCID: PMC3884855.

S2. Radial arm maze

An 8-arm typical radial maze made of plexiglass was utilized in the experiment, and a picture can be found in the Supplement. It was placed 70 cm above the floor. The maze had a central octagonal platform with a diameter of 42 cm which was surrounded by eight arms. The length of each arm was 60 cm from the center, width 17 cm, and height 25 cm. During the course of all experiments, the maze remained in a fixed position with respect to the distal cues. It was made by BSMMU and the figure is shown in the following.

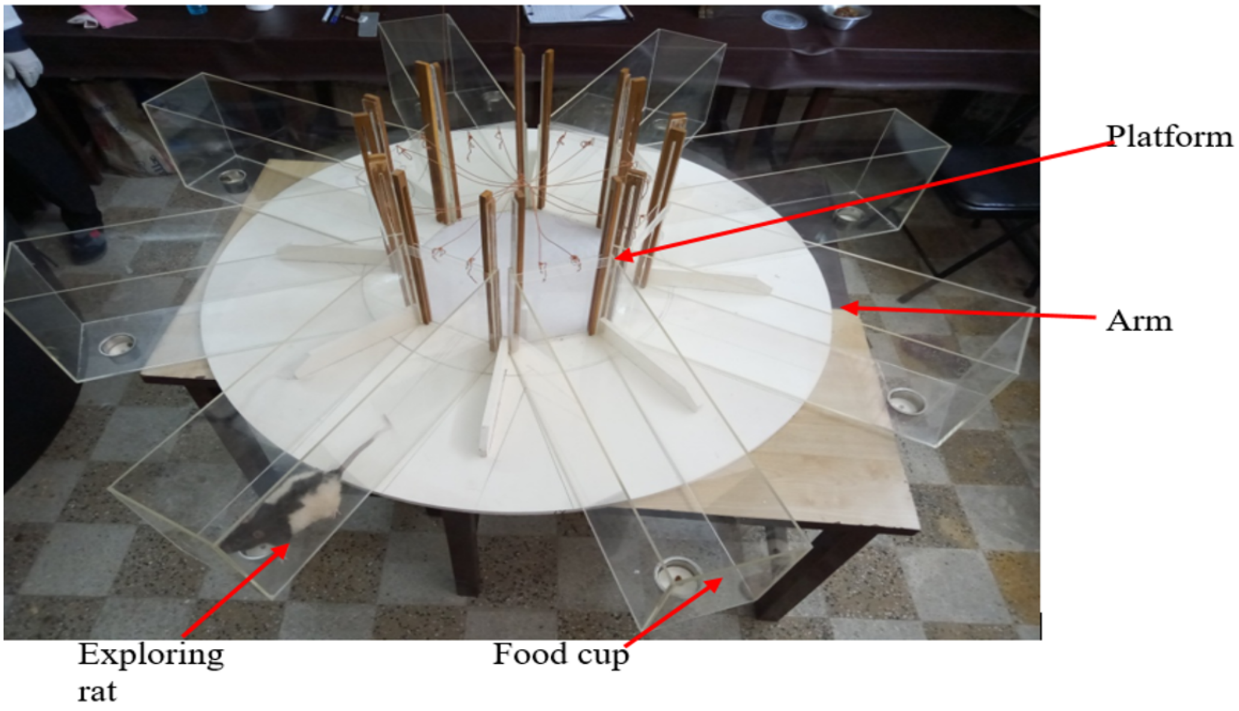

S3. Components of extract and standardization

The Process of ethanolic extraction of *Moringa oleifera* leaves was Adapted from Mahaman et al. 2018 [26].

Initially the plant leaves were collected and cleaned by fresh water. Then the leaves were dried in sunlight for 1 week. The leaves were crushed by mixer grinder and immediately stored in air tight container until use. Then the powdered leaf sample was extracted in an orbital shaker with 95% ethanol. 200 g of the MO powder was soaked in 1000ml of 95% ethanol. The extract was obtained by continuous stirring of it and allowed to keep it at room temperature for 2 days. The extract was then filtered first with a cotton plug to get rid of plant debris, and afterwards through Whatman

filter paper several times. Then it was concentrated using a vacuum rotary evaporator at 60°C.

- Quercetin, one type of flavonoid of *M. Olivera*, as *M. oleifera* is a rich source of flavonoid, was found to significantly increase the expression of NR2A and NR2B subunits of NMDARs [49]. Some researchers suggested that omega-three polyunsaturated fatty acid, a component of MO leaves, increased the NR2B subunit in the prefrontal cortex and hippocampus [50]. Some researchers also showed that the dietary polyunsaturated fatty acid of M. oleifera increased NR2A and NR2B subunits expression in the hippocampus.

Therefore, increasing the subunits of NMDARs might cause the prevention of working memory impairment in the experimental rats with *M. oleifera*.

- Ketamine was administered for five days in the acquisition phase. And ketamine was administered throughout this phase to impair animal memory and five days was recommended that can impair animal memory (Moosavi et al. 2011). In this phase, data were collected to determine group performance and the effects of ketamine and Moringa on WMEs.

S4. Plant identification, location of where they were gathered

Moringa oleifera (MO) belongs to the family of Moringacea (Mahaman et al. 2018). It is called Morunga in the Dravidian language (India), which means "generic root" (B.K and Patel 2017). Moringa oleifera is a pan-tropical species that is known by such regional names as benzolive, kelor, marango, mlonge, mulangay, nébéday, saijhan, sajna. It is commonly known as sajna in Bangladesh. English names are Horseradish tree, Drumstick tree, Never Die tree, West Indian Ben tree or Radish tree. Native to the sub-Himalayas of India, M. oleifera has been naturalized in various tropical and subtropical regions of the world, including the Middle East, Africa, the Americas, Asia, the Philippines, Cambodia, and the Caribbean islands.

This rapidly-growing tree is an edible plant. All parts of the Moringa tree are edible and have long been consumed by humans. However, it normally is cut back annually to one meter or less, and allowed to re-grow, so that pods and leaves remain within reach. The species is characterized by

its long, drumstick shaped pods that contain its seeds. Within the first year of growth, Moringa has been shown to grow up to 4 meters and can bear fruit within the same first year. The tree can grow well in humid tropics or hot dry land with 5-10 m height. The flowers are creamy white or white with 3-winged seeds in about 70 cm long grooved, cylindrical, green pods. This plant was obtained from the field of Bangladesh Council of Scientific and Industrial Research (BCSIR), Dhaka.

S5. Process of ethanolic extraction of Moringa oleifera leaves

(Adapted from Ahmed et al. 2018 and Mahaman et al. 2018)

Initially the plant leaves were collected and cleaned by fresh water. Then the leaves were dried in sunlight for 1 week. The leaves were crushed by mixer grinder and immediately stored in air tight container until use. Then the powdered leaf sample was extracted in an orbital shaker with 95% ethanol. 200 g of the MO powder was soaked in 1000ml of 95% ethanol. The extract was obtained by continuous stirring of it and allowed to keep it at room temperature for 2 days. The extract was then filtered first with a cotton plug to get rid of plant debris, and afterwards through Whatman filter paper several times. Then it was concentrated using a vacuum rotary evaporator at 60°C.
